# Displacement of a landslide retaining wall and application of an enhanced failure forecasting approach

**DOI:** 10.1007/s10346-017-0887-7

**Published:** 2017-09-05

**Authors:** Tommaso Carlà, Renato Macciotta, Michael Hendry, Derek Martin, Tom Edwards, Trevor Evans, Paolo Farina, Emanuele Intrieri, Nicola Casagli

**Affiliations:** 1 Regional Doctoral School of Earth Sciences, University of Firenze, Via La Pira 4, 50121 Florence, Italy; 2 Department of Earth Sciences, University of Firenze, Via La Pira 4, 50121 Florence, Italy; 3 Department of Civil and Environmental Engineering, University of Alberta, Donadeo ICE Building, Edmonton, T6G 1H9 Canada; 4 Canadian National Railway, Edmonton, AB Canada

**Keywords:** Landslide monitoring, Landslide retrogression, Slope deformation analysis, Retaining wall, Failure prediction, Inverse velocity

## Abstract

The 10-mile Slide is contained within an ancient earthflow located in British Columbia, Canada. The landslide has been moving slowly for over 40 years, requiring regular maintenance work along where a highway and a railway track cross the sliding mass. Since 2013, the landslide has shown signs of retrogression. Monitoring prisms were installed on a retaining wall immediately downslope from the railway alignment to monitor the evolution of the retrogression. As of September 2016, cumulative displacements in the horizontal direction approached 4.5 m in the central section of the railway retaining wall. After an initial phase of acceleration, horizontal velocities showed a steadier trend between 3 and 9 mm/day, which was then followed by a second acceleration phase. This paper presents an analysis of the characteristics of the surface displacement vectors measured at the monitoring prisms. Critical insight on the behavior and kinematics of the 10-mile Slide retrogression was gained. An advanced analysis of the trends of inverse velocity plots was also performed to assess the potential for a slope collapse at the 10-mile Slide and to obtain further knowledge on the nature of the sliding surface.

## Introduction

Adequate understanding of landslide behavior is essential for managing associated risks, and substantial information about the evolution and kinematics of a landslide can be obtained by analyzing the deformation of the slope surface (Gili et al. 2000; Brückl et al. 2006; Baldi et al. 2008; Teza et al. 2008; Sun et al. 2013). Variations in deformation trends may be the result of changes in the strength-stress regime or indicate the evolution of the moving mass toward failure (Macciotta et al. 2015). Moreover, long-term slow deformations have been repeatedly observed prior to rapid movements and a collapse of the slope (“very rapid”, as for Cruden and Varnes 1996). Displacement, strain, and velocity measurements are typically analyzed to provide early warning of potentially destructive movements. In this regard, several authors have focused on predicting the time of failure of a landslide (Crosta and Agliardi 2003; Mufundirwa et al. 2010; Federico et al. 2012; Newcomen and Dick 2015; Macciotta et al. 2015).

**Fig. 1:**
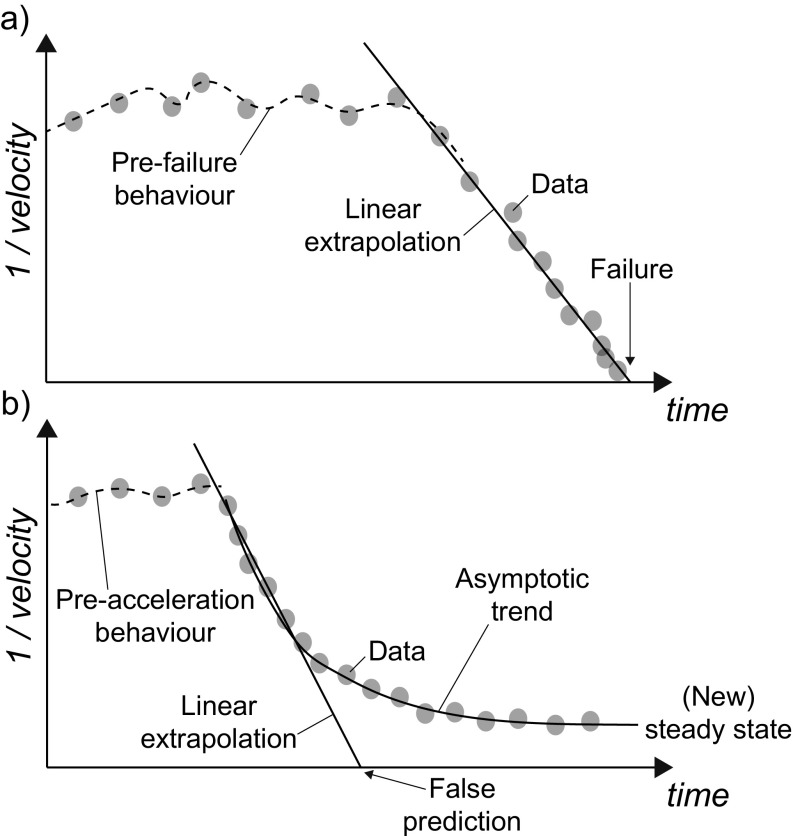
Inverse velocity trends after onset of acceleration showing **a** a linear trend leading to slope failure and **b** an asymptotic trend leading to a steady state of constant deformation

The accelerating creep theory (Saito 1969; Fukuzono 1985; Voight 1988; Voight 1989) provides the basis for many of the methodologies that analyze trends in the displacement measurements, in terms of the inverse of velocity (1/*v*, where *v* is the velocity) over time. The intercept point on the time axis of the inverse velocity vs. time plot (i.e., point of infinite slope velocity) is assumed as the failure time prediction (Fukuzono 1990; Rose and Hungr 2007) (Fig. 1a). Experience has shown that the landslide inverse velocity often displays a nearly linear trend during phases of acceleration; therefore, linear regression and extrapolation of inverse velocity data (INV in the rest of the manuscript) is commonly used as a tool to estimate the time of slope failure.

There are implicit simplifications and assumptions on which the accelerating creep theory is based that hamper the reliability of failure time predictions (Fell et al. 2000; Rose and Hungr 2007; Federico et al. 2012). As a result, INV does not allow for the prediction of the exact time of failure, and its application only indicates that failure is likely in proximity of the point of intersection of the extrapolated linear inverse velocity trend with the time axis. The observation of a linear inverse velocity trend does not always imply failure: the slope may progressively decelerate, as it reaches a new condition of stability (i.e., “regressive deformation”, Zavodni and Broadbent 1980) or evolve to a constant rate of deformation (Fig. 1b). In the latter case, the inverse velocity trend becomes asymptotic to the time axis. As long as such trend persists, even if rates of slope deformation are high, it is typically not possible to perform predictions with the time axis intercept, as there is no longer an intercept find, or this is so far in the future that there is very low confidence in the extrapolated values of failure time.

**Fig. 2:**
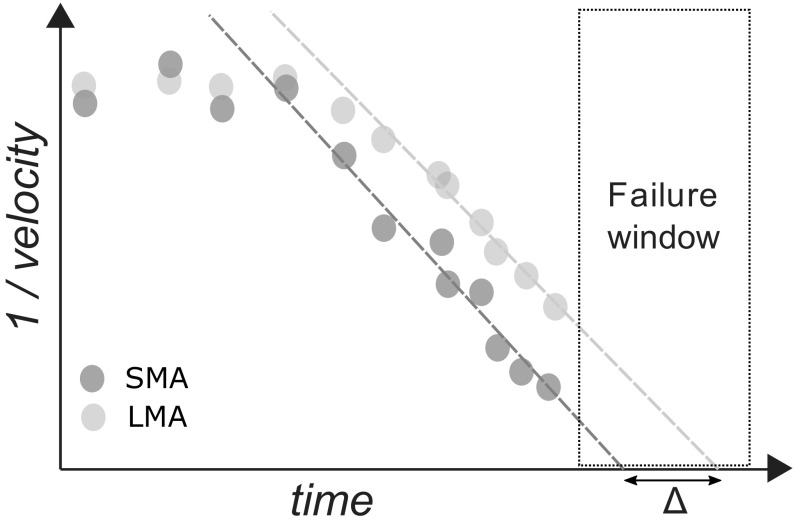
Conceptual model of failure window analysis: SMA represents displacement data filtered by means of a short-term moving average and LMA by means of a long-term moving average. The width of the failure window is calculated on the basis of the Δ between the LMA and SMA failure time predictions

Following these considerations and after evaluating also the effects of instrumental noise on inverse velocity analyses, Carlà et al. (2016) proposed an approach to define the time interval during which the occurrence of a failure event may be expected (“failure window,” *T*_*fw*_). Such approach consists in smoothing monitoring data by means of both a short-term (SMA) and a long-term moving average (LMA). After projecting simultaneously the linear best-fits of the SMA and LMA inverse velocity series, *T*_*fw*_ is obtained on the basis of the difference Δ between the SMA failure time prediction (*T*_*f*(*SMA*)_) and the LMA failure time prediction (*T*_*f*(*LMA*)_, Fig. 2). The appropriate width of the failure window may vary from case to case and depends on several different factors, such as frequency of monitoring data, landslide deformation characteristics, accuracy of the measurements, and level of tolerable risk.

Carlà et al. (2016) described the successful application of this method in four case histories that showed typical acceleration prior to failure. Recorded time of failure for each case was within the failure window, which was defined as:

1 $$ {T}_{fw}=\left[{T}_{f(SMA)}-\frac{\varDelta }{2};{T}_{f(LMA)}+\frac{\varDelta }{2}\right] $$

where *Δ* = *T*_*f*(*LMA*)_ − *T*_*f*(*SMA*)_. The SMA and LMA filters used for smoothing the velocity at time *t* are given by:

2 $$ {\overline{\mathrm{v}}}_{\mathrm{t}}=\frac{{\mathrm{v}}_{\mathrm{t}}+{\mathrm{v}}_{\mathrm{t}-1}+\dots +{\mathrm{v}}_{\mathrm{t}-\left(\mathrm{n}-1\right)}}{\mathrm{n}} $$

where ${\overline{\mathrm{v}}}_{\mathrm{t}}$ is the smoothed velocity at time *t*, and with *n* = 3 and *n* = 7 for the SMA and LMA, respectively. Although the method has proven successful for cases where slope failure was anticipated by increased accelerations, it was among the aims of this study to stress-test it against a case showing periods of acceleration and deceleration.

This paper presents the review of the displacement measurements at a railway retaining wall within the 10-mile Slide in Canada. Design and construction at the site were undertaken by third parties under contract with the Canadian National Railway (CN). Wall monitoring data, which were also acquired and provided by CN as part of the safety management system, were validated as representative of the soil mass. The 10-mile Slide is in fact a translational landslide that shows a discrete, basal zone of shear, with no differential movement within the moving mass. Retrogression of the landslide extended the shear surface below the bottom of the pile wall. Moreover, displacements at the tip of the piles have been consistent with displacements measured by subsurface instrumentation. Unfortunately, subsurface instruments shear quickly due to the landslide displacement rates. Therefore, displacements of the pile tips have been considered representative of landslide displacement in the vicinity of the railway tracks, and adequate for enhancing the risk control measures in place.

In the following sections, the 10-mile Slide is described, and the displacement measurements provided by CN are then used to analyze its deformation trends and to investigate the application of the forecasting approach proposed by Carlà et al. (2016). The case study showed that thorough insights into the behavior and kinematics of landslides may be gained by monitoring the deformation of structures involved in the instability.

## The 10-mile Slide

**Fig. 3:**
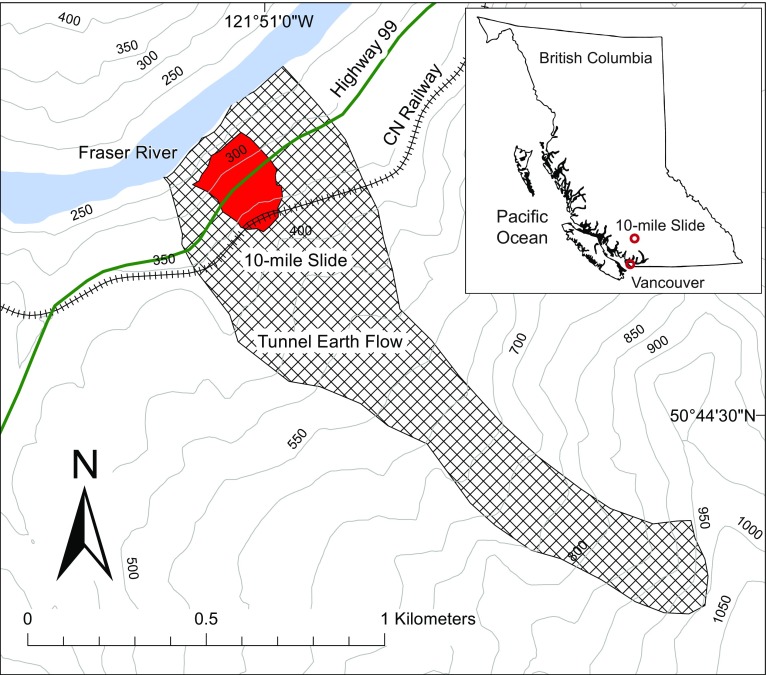
Location of the 10-mile Slide within the province of British Columbia and plan view of the landslide relative to the older, larger Tunnel earthflow. Highway 99 and CN’s railway are also shown (after Macciotta et al. 2017a; Bovis 1985)

**Fig. 4:**
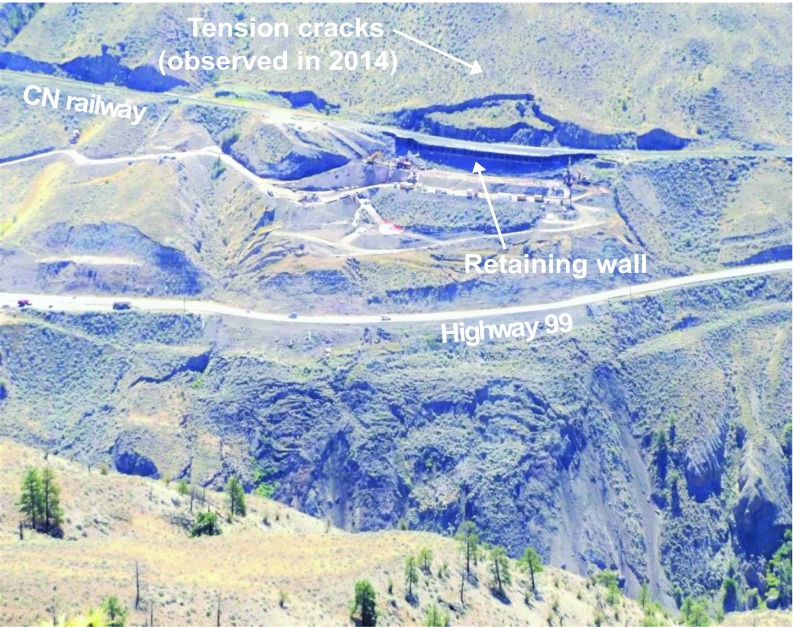
Front view of the 10-mile Slide. Photo taken in July 2016

The 10-mile Slide is located in the Province of British Columbia, Canada, North of the town of Lillooet (Fig. 3). The slide is an active portion of the ancient Tunnel earthflow described by Bovis (1985), and its name is related to its location (10th mile board of Highway 99). A section of British Columbia’s Highway 99 and a section of railway operated by CN cross through the landslide boundaries. The landslide and the retaining structures have been described before in Gaib et al. 2012 and in Macciotta et al. 2017a, b. The significance of the landslide is associated with the integrity of these structures, which connect localities in the interior of British Columbia. Figure 4 shows a front view of the landslide, as of July 2016.

The 10-mile Slide has been monitored for over 40 years. In this period, observed deformation velocities have reached 10 mm/day (Gaib et al. 2012). These landslide deformations have been concentrated downslope of the railway alignment and have affected the integrity of the highway. Landslide effects to the railway operations have been managed through scheduled maintenance, track geometry measurements, and a visual inspection of the track before each train passes. Also, a retaining wall was installed immediately downslope from the railway track in 2008 to delay retrogression of the landslide and minimize the impact of the downslope deformations on rail operations; as such goals were no longer effectively met and significant landslide movements were measured again starting from January 2015, further stabilization works were completed by October of 2016.

### Geology and climate at the study area

The 10-mile Slide is located in an area of the Canadian Cordillera where peaks reach over 2000 m in elevation. The landslide is within the Fraser River valley, characterized by a U-shaped cross-section typical of glacial erosion. Glacial and post-glacial sediments are common along the valley slopes and bottom. The Fraser River has further incised the valley bottom, over-steepening the valley slopes.

The bedrock lithology in the area includes andesite to dacite volcanic rocks, sandstone, and shale. These rocks are overlain by quaternary deposits (including glacial drift blankets, colluvium, alluvium, and landslide deposits) (McTaggart and Thompson 1967; Bovis 1985).

Weather records for Lillooet for the years 1985 through 2014 report an annual average precipitation of about 350 mm/year. Temperatures below 0° are common between November and March (Environment Canada 2014).

The 10-mile Slide is a reactivated portion of a larger, post-glacial earth flow named Tunnel earthflow (Fig. 3). This earth flow is considered inactive with the exception of the portion of the 10-mile Slide. The Tunnel earthflow has a complex stratigraphy that includes clayey-sheared zones and volcanic material interlayered with poorly lithified sediments (Bovis 1985). This is reflected in a very heterogeneous profile throughout the area of the 10-Mile Slide (Gaib et al. 2012).

### Dimensions, stratigraphy, and kinematics

**Fig. 5:**
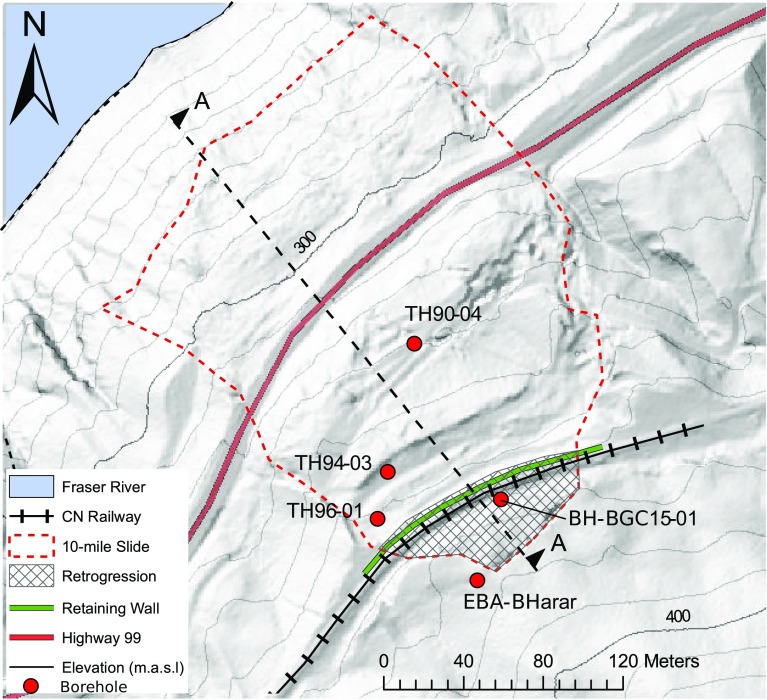
Plan view of a shaded relief of the 10-mile Slide. Shaded relief corresponds to 2015 LiDAR imaging (after Macciotta et al. 2017a)

A plan view of the shaded relief of the 10-mile Slide is shown in Fig. 5. The shaded relief was derived from a digital elevation model (1 m resolution) obtained in 2015 through aerial Light Detection and Ranging (LiDAR) provided by CN.

The width and length of the actively deforming mass are about 200 and 260 m, respectively. Landslide depth is 20 m in average, as determined from borehole surveys (see later in this Section). The approximate volume of the landslide is between 750,000 m^3^ (as first reported in Gaib et al. 2012) and 1 × 10^6^ m^3^, as estimated following some retrogression observed between 2012 and 2016. Since the 1980s, displacement rates have varied spatially and temporally, with recorded rates up to 10 mm/day. The area of deformation has been inferred from sharp scarps and tension cracks, most of them visible in the shaded relief (Fig. 5). Mapping of the landslide features was obtained through a combination of detailed digital delineation based on the observable topographical attributes on the digital elevation model and surface in situ mapping.

**Fig. 6:**
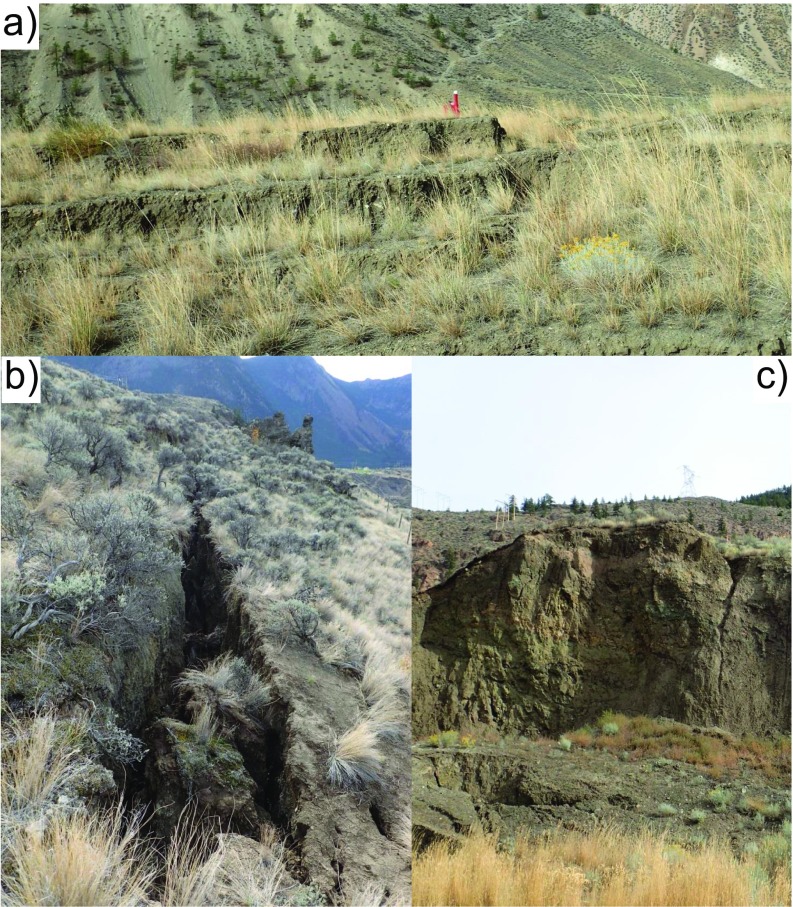
Counter-slope scarps located between the railway track and Highway 99 (**a**), the uppermost tension crack observed above the railway track, as of April 2016 (**b**), and an 8- to 10-m-high scarp downslope from the railway track (**c**)

Figure 6 presents images of counter-slope scarps located between the railway track and Highway 99 (a), the back scarp observed above the railway track as of April 2016 (b), and an 8- to 10-m-high scarp downslope from the railway track (c). Tension cracks have been observed upslope of the back scarp in April 2016, suggesting stress relief of these areas and potential further retrogression.

Borehole investigations since 1989 suggest that the stratigraphy in the area of the landslide consists of a layer (up to 20 m thick) of mixed landslide deposits including medium to high plastic clays, silts, and the presence of zones of varying sand and gravel content. This layer overlies colluvium materials (sand, silt, and clay), which in turn overly glacial deposits (stiff, gravelly clayey, and silty till with the presence of glaciofluvial well graded, rounded gravel, and sand deposits) (BGC Engineering Inc. 2015). However, the area presents a heterogeneous soil profile, highly variable between borehole locations (Gaib et al. 2012). The position of the boreholes is depicted in Fig. 5.

**Fig. 7:**
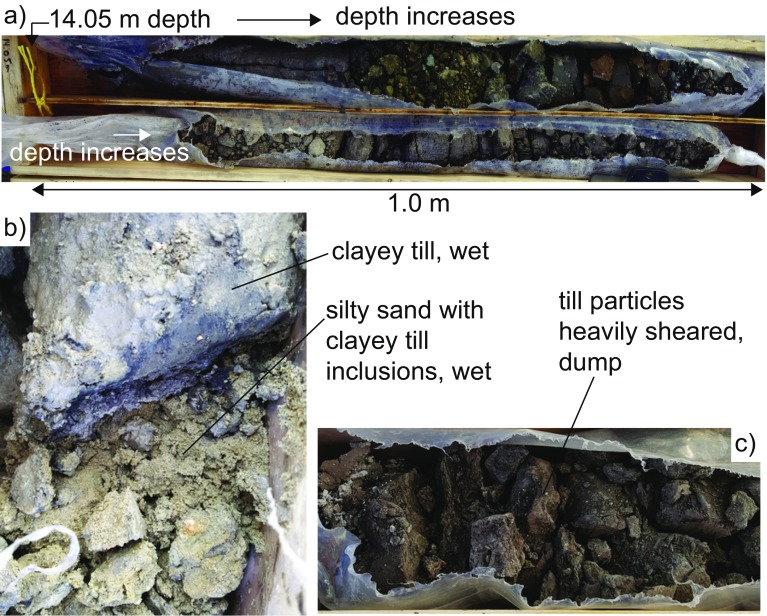
Core material recovered from the landslide area at a depth between 14 and 16 m from the surface (**a**), detail of a sharp change between wet clayey till cohesive core and silty sand material with till inclusions (**b**), and core with heavily sheared till (**c**). After Macciotta et al. (2017b)

Figure 7 shows core material recovered in 2015 from the landslide area (Borehole BH-BGC15-01), in the vicinity of the railway track. The core corresponds to a depth between 14 and 16 m from the slope surface. This recovered core is highly variable within the landslide (clays, gravels, and stiff silts can be observed within less than 2 m).

**Fig. 8:**
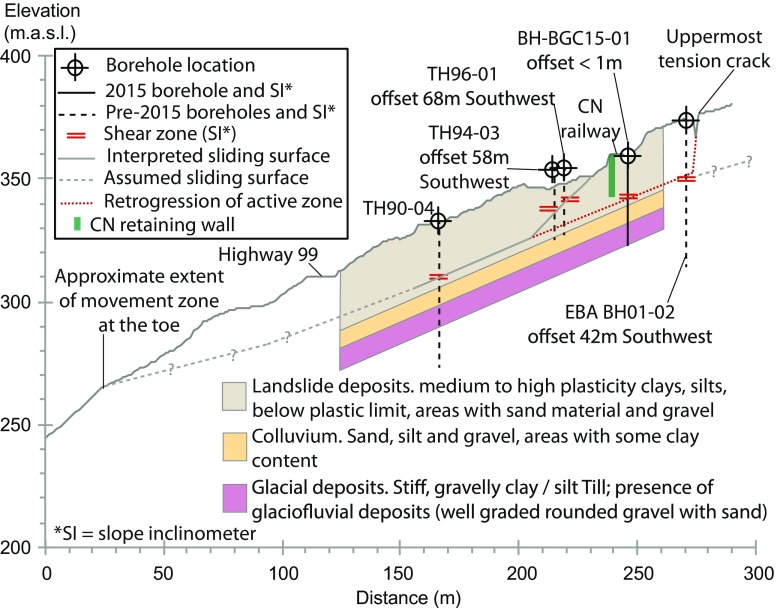
Interpreted simplification of the cross-section of the 10-mile Slide (after Macciotta et al. 2017a)

A number of slope inclinometers (SI) have been installed in the area (Gaib et al. 2012). These were typically sheared within weeks of installation due to the deformation velocities. Readings from these SI, and in particular, an SI installed in September of 2015 (BGC Engineering Inc. 2015) suggest that the 10-mile Slide is sliding on a through-going shear surface, extending upslope beyond the location of CN’s railway. This through-going shear surface appears to have a dip of about 23°, which is essentially parallel to the ground surface. Figure 8 shows a simplified interpretation of the stratigraphy of the 10-mile Slide along Fig. 5a. The SI installed immediately upslope of CN tracks in September of 2015 (BH-BGC15-01 in Fig. 8) showed a depth of sliding consistent with the through-going rupture surface inferred for the lower portions of the landslide. Displacement rates measured with this SI were between 5.2 and 5.9 mm/day for the period of September 16 through September 22, 2015. An SI in borehole EBA-BH01-02 (Fig. 5) was located further upslope of the tracks to monitor potential retrogression of the landslide. Initially, this SI did not show signs of retrogression. This instrument responded during the latest phases of displacement shown in this paper, with an average velocity of 28 mm/year in early 2014, suggesting that retrogression of the landslide had been initiated beyond the mapped boundaries.

Vibrating wire piezometers installed at different depths within the boreholes at the 10-mile Slide have measured pore water pressures equal to or less than atmospheric pressure. It was concluded that excess pore pressures were not the driving mechanism for slope movements; however, increased shear strengths due to suction would play a role in the stability of the landslide. Slope movement trends have not been observed to correspond to excessively dry periods or with sustained wet periods of precipitation events. This suggests that weather effects would not affect significantly the landside stability in the short term. Landslide continuous displacement is attributed to the anthropogenic activity in the area, together with constant geometric change of the slope mass following the sliding creep behavior through the basal shear surface.

### Retaining wall

**Fig. 9:**
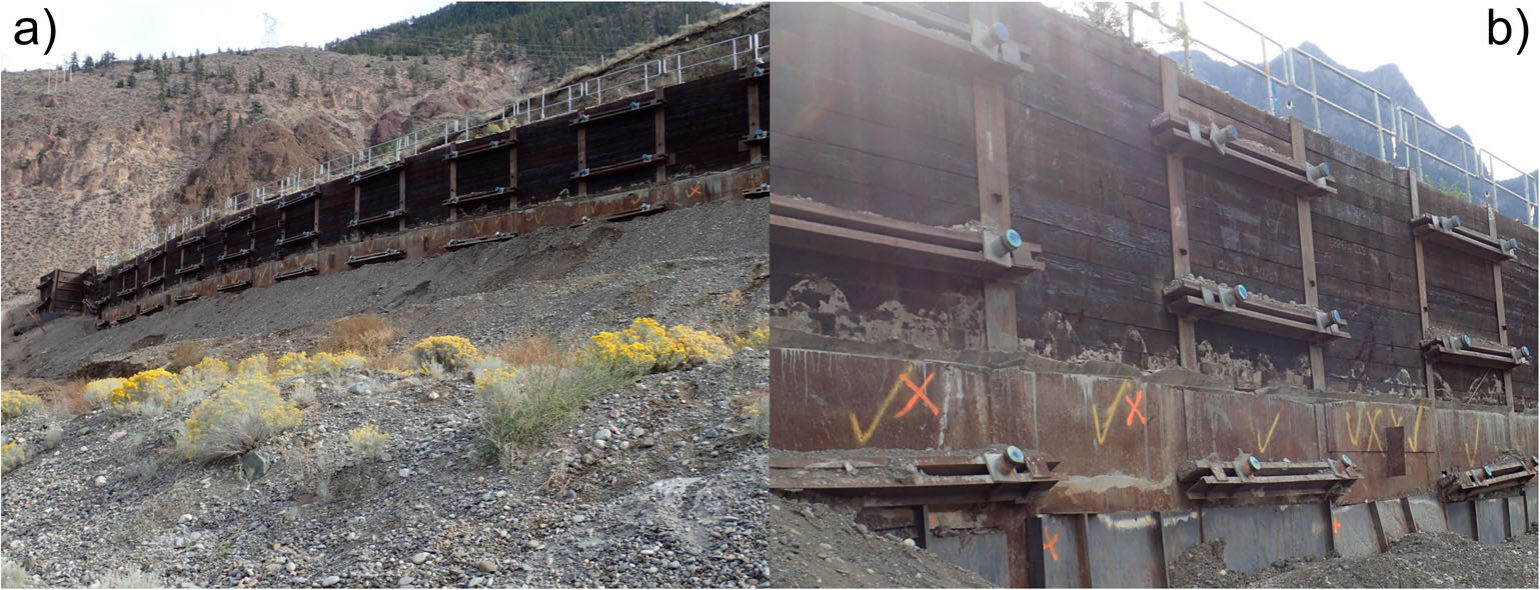
View of the retaining wall from the northernmost area of the landslide (**a**) and close view of the anchoring system (**b**)

A retaining wall was installed in 2008 and was later extended in 2010 (Fig. 9). The wall was installed to prevent deformations caused by potential loosening of materials associated with the slope deformations and to delay possible landslide retrogression. The retaining wall is 128-m-long and consists of 43 H-piles driven to refusal, approximately 16.5 m into the ground, separated 3.05 m (10 ft), and with lagging in between. Three rows of 12-m-long anchors provide the active resistance of the wall (close view of the anchoring system in Fig. 9b). Depth of piles and anchors are above the sliding surface. In response to the reactivation of the landslide movements, 253 new shear piles were installed approximately between 20 June and 7 October 2016.

## Monitored displacement trends

**Fig. 10:**
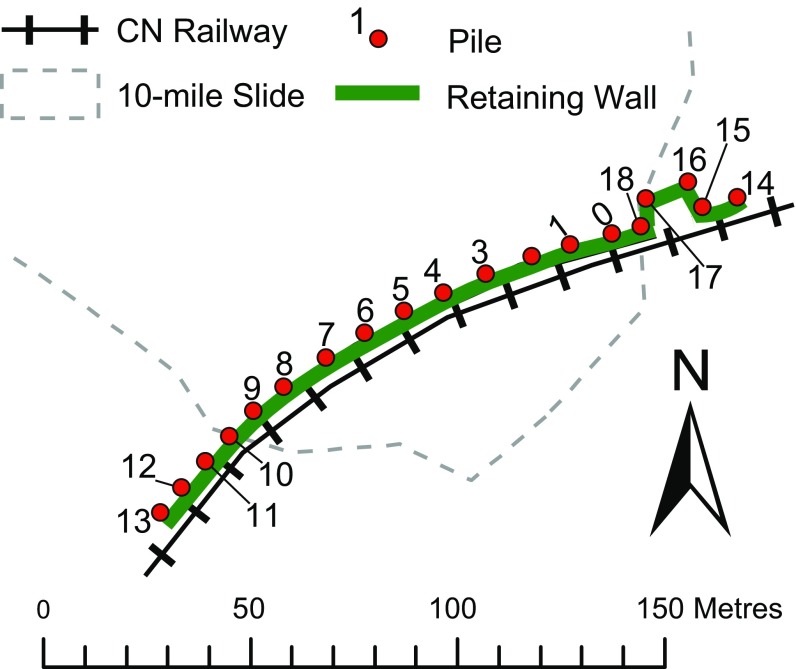
Sketch with the location of the retaining wall piles being monitored for displacement

Surveyed monitoring of the retaining wall began in 2011 and is conducted with a total station and prisms installed on 19 of the piles of the retaining wall. The prisms were located at the top of the piles, and the monitoring station was located northeast of the landslide site, about 300 m from the wall. A plain view with the location of the piles within the wall is shown in Fig. 10. Monitoring was carried out by a third party survey company. The station was considered stable relatively to the landslide displacement rates, and it was regularly checked as part of the surveyors QA/QC. For the distances between prisms and the total station, the expected single measurement accuracy was ± 2 − 3 mm. This was considered adequate for the large displacement rates observed in the field. Initially, monitoring was performed with quarterly measurements (in average) and was then progressively enhanced up to weekly measurements by July–August 2015, reflecting the acceleration of the landslide and the perceived hazard levels. No significant pile displacements were recorded until early 2015, when displacement rates started to increase. Monitoring at Pile 17 had technical difficulties and was stopped prematurely; thus, no information was obtained for this pile.

Of the 19 piles, 10 of them (piles 0–9, or “central piles”) are definitely identified within the boundaries of the landslide movement, whereas the others (piles 10–18, or “lateral piles”) are located on or just outside such boundaries (Fig. 10).

The characteristics of the movements of the central piles are markedly different from those of the lateral piles. Conversely, the characteristics of the movements are extremely similar among piles of the same group. In the following sections, in cases where for illustration purposes it is not convenient to show data for all piles, piles 0 and 6 represent the characteristics of the movements of the central piles, while piles 13 and 14 represent the characteristics of the movements of the lateral piles.

### Cumulative displacement and displacement velocity

The surveyed data consist of the 3-dimensional coordinates of each pile prism for each monitoring date. Incremental displacements ${\widehat{d}}_{i(X)}$, ${\widehat{d}}_{i(Y)}$, and ${\widehat{d}}_{i(Z)}$ for the period between time *t*_*i* − 1_ and time *t*_*i*_ correspond to movements in the North, East, and elevation directions, respectively. These are calculated as the difference in position between *t*_*i* − 1_ and *t*_*i*_. The horizontal component of the incremental displacement at time *i* is calculated according to:

3 $$ {\overset{\hat{\mkern6mu} }{d}}_{i(h)}=\sqrt{{\left({\overset{\hat{\mkern6mu} }{d}}_{i(X)}\right)}^2+{\left({\widehat{d}}_{i(Y)}\right)}^2} $$

while the vertical component of incremental displacement at measurement *i* is directly derived from ${\widehat{d}}_{i(v)}={\widehat{d}}_{i(Z)}$.

Consequently, cumulative displacements at time *i* are calculated as:

4 $$ {D}_{i(h)}={\sum}_{j=1}^i{\overset{\hat{\mkern6mu} }{d}}_{j(h)} $$

5 $$ {D}_{i(v)}={\sum}_{j=1}^i{\overset{\hat{\mkern6mu} }{d}}_{j(v)} $$

**Fig. 11:**
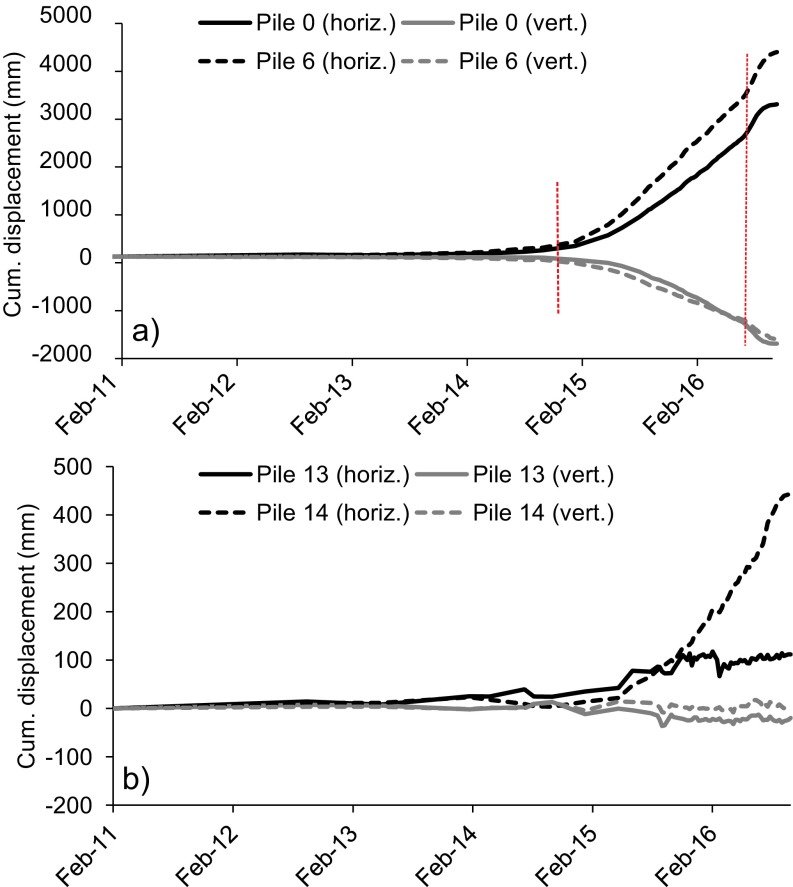
Time series of cumulative horizontal and vertical displacement of piles 0 and 6 (**a**), and of piles 13 and 14 (**b**) from February 2011 to September 2016. The two red dashed lines mark the onset of the first and second acceleration phase, respectively

Figure 11 depicts the measured cumulative horizontal and vertical displacements of selected piles between February 2011 and September 2016. The central piles show persistent displacements from January 2015 up to September 2016, with total displacements ranging between 2.5 and 4.5 m horizontally and between 1.5 and 2.1 m vertically. Cumulative displacement acceleration of these central piles was observed in January 2015 and June 2016 (red dashed lines in Fig. 11a). While the first acceleration phase reflected the natural dynamics of the landslide, the second acceleration phase was associated with the installation of the new shear piles (see “Retaining wall” Section); this in fact required drilling and consequently determined a temporary increase of pore water pressure, which prompted acceleration of the landslide. On the other hand, movement of the lateral piles is about one order of magnitude smaller and in most instances does not appear to be consistent over time (Fig. 11b—note the scale is amplified by a factor of 10 with respect to Fig. 11a).

**Fig. 12:**
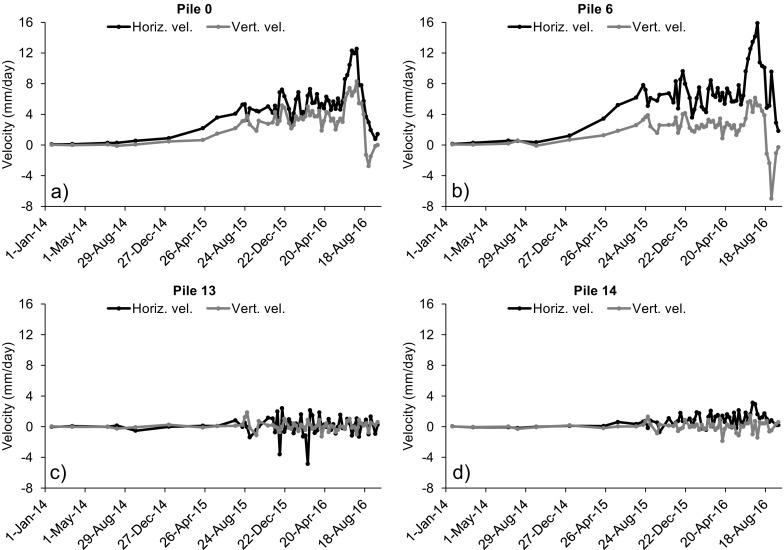
Velocities of piles 0 (**a**), 6 (**b**), 13 (**c**), and 14 (**d**) from February 2011 to September 2016

Figure 12 illustrates the corresponding displacement velocities of the piles in Fig. 11, in millimeters per day. The discrepancy between the behavior of central and lateral piles results evident: the central piles show the two aforementioned distinct phases of acceleration, separated by a prolonged phase of mostly constant velocity which is characterized by values ranging from approximately 3 to 9 mm/day horizontally and from 2 to 4 mm/day vertically. A peak velocity of 15.9 mm/day in the horizontal direction is identified on 25 July 2016 for pile 6. Afterwards, velocities started to decrease and ceased to be significant at the end of September 2016, when the stabilization works were being finalized. Conversely, the lateral piles never showed a consistent phase of acceleration in the dataset. Starting from August 2015 velocities were typically scattered between 0 and 3 mm/day in the horizontal direction and between 0 and 1 mm/day in the vertical direction (i.e., within the interval of measurement accuracy).

### Evolution of displacement trends

Survey of the piles coordinates allowed also calculation of the direction of horizontal and vertical movement, expressed in terms of azimuth (*α*) and dip angle (*β*). The azimuth angle (°) of the horizontal component of movement at measurement *i* is calculated according to:

6 $$ {\alpha}_i=\arctan \left({\overset{\hat{\mkern6mu} }{d}}_{i(X)}/{\overset{\hat{\mkern6mu} }{d}}_{i(Y)}\right) $$

Similarly, the dip angle (°) of the displacement vector at measurement *i* is:

7 $$ {\beta}_i=\arctan \left({\overset{\hat{\mkern6mu} }{d}}_{i(h)}/{\overset{\hat{\mkern6mu} }{d}}_{i(v)}\right) $$

Differently, from its typical use for mapping planar features in engineering geology applications, the dip angle is here considered to vary between 0° and 360°, with a value of 0° indicating a perfectly horizontal movement out of the slope, and a value of 180° a perfectly horizontal movement toward the slope; consequently, a 90° dip angle indicates a perfectly vertical downward movement, while a 270° dip angle a perfectly vertical upward movement.

**Fig. 13:**
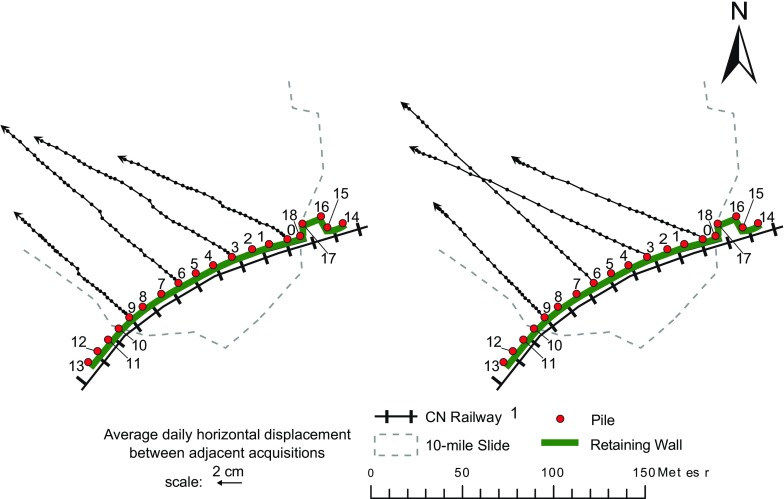
Increments of horizontal displacement of selected central piles from January 2015 to March 2016 (left) and from April to September 2016 (right)

**Fig. 14:**
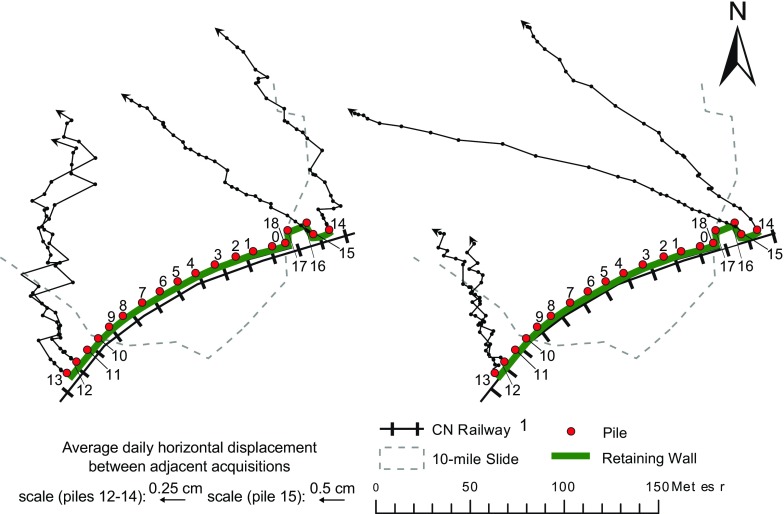
Increments of horizontal displacement of selected lateral piles from January 2015 to March 2016 (left) and from April to September 2016 (right)

The geometrical characteristics of the displacements of the piles and their evolution can then be analyzed. Figures 13 and 14 show the direction of the increments of horizontal movement (azimuth) of selected central and lateral piles from January 2015 (start of the landslide deformation) to September 2016 (end of the landslide deformation due to completion of the stabilization works). In the mentioned figures, such time interval is divided into two segments for illustration purposes. The length of the vector increments is proportional to the average daily horizontal displacement for the relative interval of monitoring (different scales in Figs. 13 and 14 were also used for illustration purposes). Figure 13 indicates that the direction of horizontal movement of the central piles was mostly constant throughout the entire period of landslide deformation. The azimuth is generally sub-parallel to the aspect of the slope; however, in the eastern sector of the landslide (see piles 0 and 3), this is slightly shifted toward the central part of the landslide (see pile 6). This can be explained by the dragging action of the central section of the wall, which has suffered the largest displacements and thus pulls the other piles toward the center.

Conversely, the lateral piles (Fig. 14), and in particular those located outside the western boundary of the landslide (see piles 12 and 13), were characterized by a much more variable-measured direction of horizontal movement. In the last part of the monitoring period, pile 15 appears to assume more consistent azimuth values, similar to those of pile 0 (Fig. 13). However, the intensity of the displacements was not as significant. The scale in Fig. 14 is different than in Fig. 13 in order to magnify the increments of piles displacement; moreover, the scale of piles 12–14 is further amplified with respect to that of pile 15 to better appreciate the changes in movement direction.

**Fig. 15:**
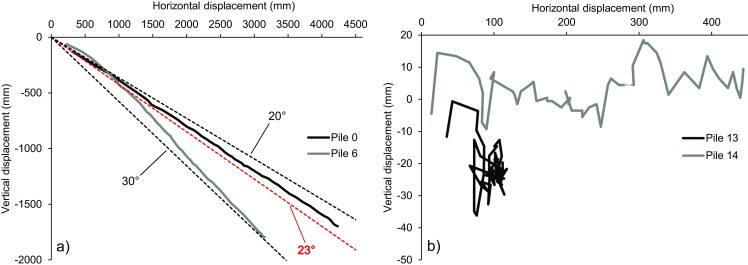
Horizontal vs. vertical movements between January 2015 and September 2016 of piles 0 and 6 (**a**), and of piles 13 and 14 (**b**). In **a**, the black dashed lines represent movement over ideal surfaces of 20° and 30°, whereas the red dashed line over an ideal 23° surface

Similar observations can be made by analyzing the trend of cumulative horizontal vs. vertical displacement of the piles for the period January 2015 to September 2016 (Fig. 15). At the central piles, a mainly constant relation between horizontal and vertical movements can be appreciated. The inclination of the line plots does not vary significantly with time and is at an angle close to 23°, consistent with the estimated inclination of the basal sliding surface. This was expected for a landslide sliding over a basal, planar surface. On the other hand, the erratic behavior shown by the lateral piles correspond to processes of landslide retrogression combined with the dragging action of the wall. As the landslide moves downslope, it drags the center of the pile wall, which in turn drags the lateral extents. Since movements of the lateral piles are much more limited with respect to the central piles, the mentioned variability may in part also be a consequence of measurement error constituting a larger percentage of the measured data.

**Fig. 16:**
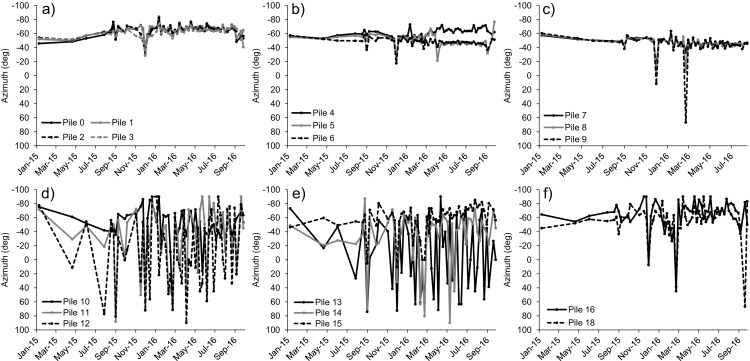
Evolution of the azimuth angle of movement with time of the piles of the railway retaining wall. An azimuth of 0° indicates a perfectly northward direction of movement

**Fig. 17:**
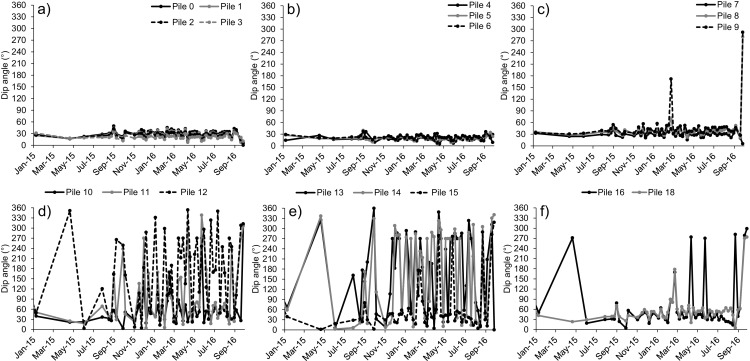
Evolution of the dip angle of movement with time of the piles of the railway retaining wall. A dip angle of 0° indicates a perfectly horizontal movement away from the slope

Additional details on the geometry of the wall movements can be determined in Figs. 16 and 17, where the azimuth and dip angle of each increment of displacement of every pile since January 2015 to September 2016 are reported. Except for a few spikes, the characteristics of the movement of the central piles (Fig. 16a–c and Fig. 17a–c) remained consistent throughout the entire landslide deformation phase, with values of azimuth typically ranging between − 40° and − 70° with respect to the North (i.e., approximately in the NW-NNW direction) and values of dip angle ranging between 15° and 30°. The measured azimuth and dip angles of movement of the lateral piles (Fig. 16d–f and Fig. 17d–f) were instead extremely variable, with displacements ranging between − 90° and 90° with respect to the North (i.e., from West to East) and in both downward and upward direction. As previously mentioned, this marked variability may be explained with the dragging action of the wall and with the higher impact of measurement error at piles characterized by low displacements.

As a result, the piles of the railway retaining wall at the 10-mile Slide can be classified according to two types of deformation behavior:

- Type 1 (central piles): characterized by large overall displacements (several meters in horizontal direction), consistent phases of progressive acceleration, and low variability of the azimuth and dip angles of movement.
- Type 2 (lateral piles): characterized by lower overall displacements (< 1 m in horizontal direction), lack of consistent phases of progressive acceleration, and high variability of the azimuth and dip angles of movement.

**Fig. 18:**
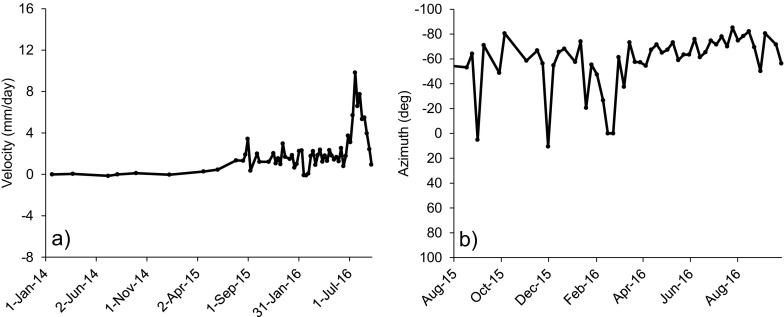
**a** Horizontal velocity and **b** variation of azimuth angle with time of pile 15, showing a transition from Type 2 to Type 1 deformation behavior

In relation to the ongoing maintenance of the wall, in the latest part of the monitoring period, a change from Type 2 to Type 1 deformation behavior was observed concerning some of the piles located in proximity of the eastern boundary of the landslide. As mentioned preliminarily in Fig. 14, this resulted evident in particular in the case of pile 15, which displayed a progressive increase in velocity in July 2016 (values of up to 9 mm/day in horizontal velocity) and a stabilization of the measured azimuth of movement starting from April 2016 (Fig. 18). This suggests that the boundaries of the 10-mile Slide are subject to further phases of active retrogression.

## Inverse velocity and forecasted time to failure

Since the characteristics of the 10-mile dataset (e.g., range of acquisition frequency and accuracy of the measurements) are comparable to the velocity time series analyzed by Carlà et al. (2016), the failure window was herein defined according to the same criteria (Eqs. 1 and 2). The 10-mile Slide has experienced persistent and intense deformation starting from January 2015 to September 2016: two main episodes of acceleration occurred along such period, the second one being determined by anthropogenic causes (i.e., drilling related to the installation of 253 new shear piles). As described in Fig. 12 and in “Cumulative displacement and displacement velocity” Section, the first acceleration phase coincides with the initial reactivation of the landslide movements in January 2015. The importance of the second acceleration phase is associated with the evidently larger velocities with respect to what previously observed (these were also the largest velocities measured in the entire time interval of the monitoring data). In fact, variations in velocity prior to June 2016 are quite regularly scattered around a somewhat constant value and thus have a generally horizontal trend, whereas the acceleration starting in June 2016 is clearly spiking above such trend and is more consistent with time (Fig. 12). An event of catastrophic failure ultimately did not occur, and displacements subsided at the end of September 2016 as the stabilization works were being completed.

The *T*_*fw*_ approach was thus applied to the 10-mile dataset in order to account for the implicit uncertainty of INV analyses, to evaluate the risk that was posed by periods of acceleration (i.e., how far in time the 10-mile Slide was from theoretical infinite velocity) and to investigate whether false alarms would have been in place; to this aim, the two described phases of acceleration were considered, as these would most likely trigger alarms during monitoring and early warning. Ultimately, this also allowed assessing the adequacy of the method to this particular case study.

**Fig. 19:**
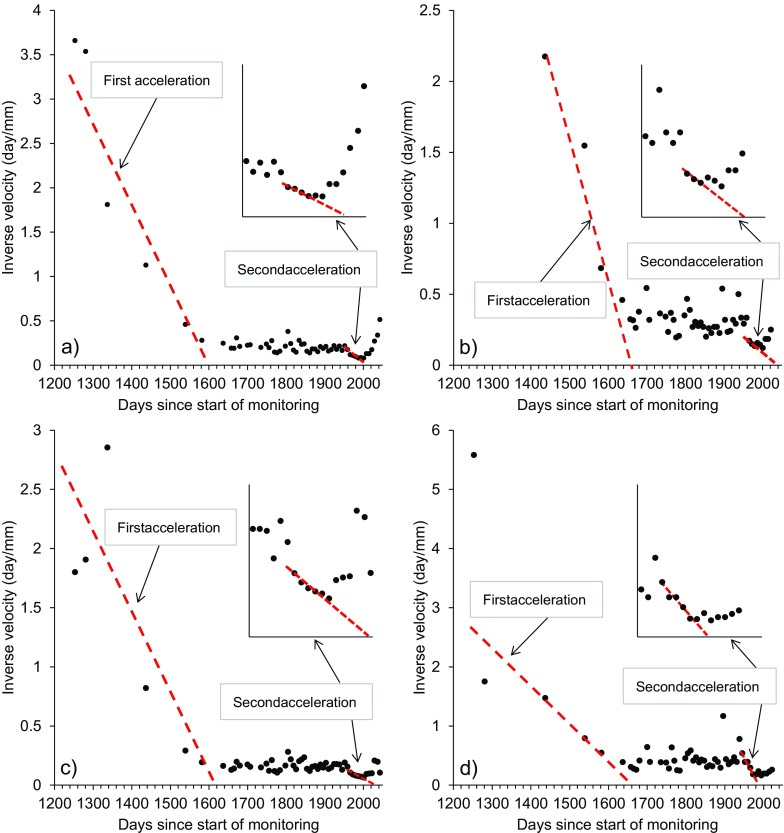
**a** Plot of inverse horizontal velocity of pile 0. **b** Plot of inverse vertical velocity of pile 0. **c** Plot of inverse horizontal velocity of pile 6. **d** Plot of inverse vertical velocity of pile 6. All plots are based on unfiltered data and highlight the two main landslide acceleration phases that occurred during the monitoring period.

The accelerations can be appreciated in the displacement time series of every central pile; therefore, use of the corresponding inverse velocity plots permits evaluation of the nature of the trend toward failure of the 10-mile Slide. In Fig. 19, both the horizontal and vertical inverse velocity plots of piles 0 and 6 are shown to exemplify the behavior of the central piles: even if a certain amount of noise affects the unfiltered data (especially concerning movements in the vertical direction, which in fact were of lower intensity), the points of trend change (*T*_*c*_) marking the end of the two acceleration phases are identified for every central pile on day no. 1582 and day no. 1980 since the start of monitoring. These correspond to 2 June 2015 and 4 July 2016, respectively. In each case, the inverse velocity trend, linear in the initial part during the first acceleration phase, becomes markedly asymptotic with respect to the time axis after the first point of trend change (steady state of constant deformation, see “Introduction” Section). Conversely, the point of trend change identifying the end of the second acceleration phase marks the start of the deceleration which ended at the end of September 2016, when significant landslide movements were no longer measured.

**Fig. 20:**
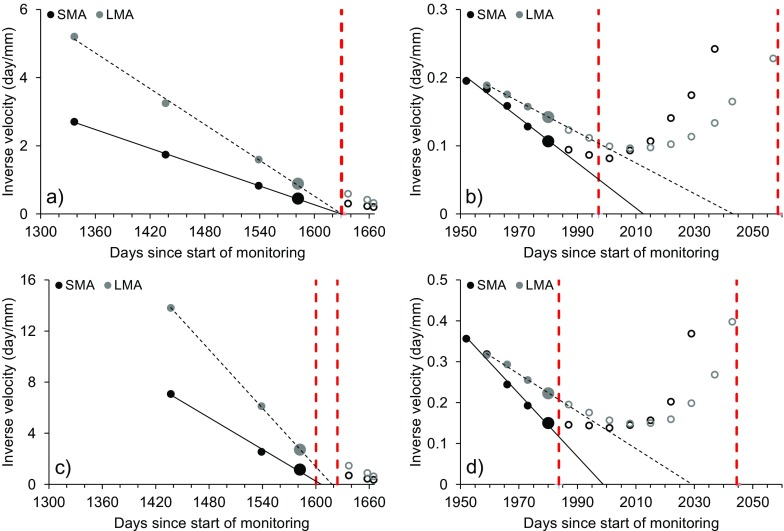
Failure window approach applied to inverse velocity data of pile 0. **a** First acceleration phase, horizontal direction. **b** Second acceleration phase, horizontal direction. **c** First acceleration phase, vertical direction. **d** Second acceleration phase, vertical direction. The red dashed lines define the limits of the failure window, the larger points mark days no. 1582 and 1980 (i.e., *T*_*c*_), and the hollow points represent measurements after *T*_*c*_

**Fig. 21:**
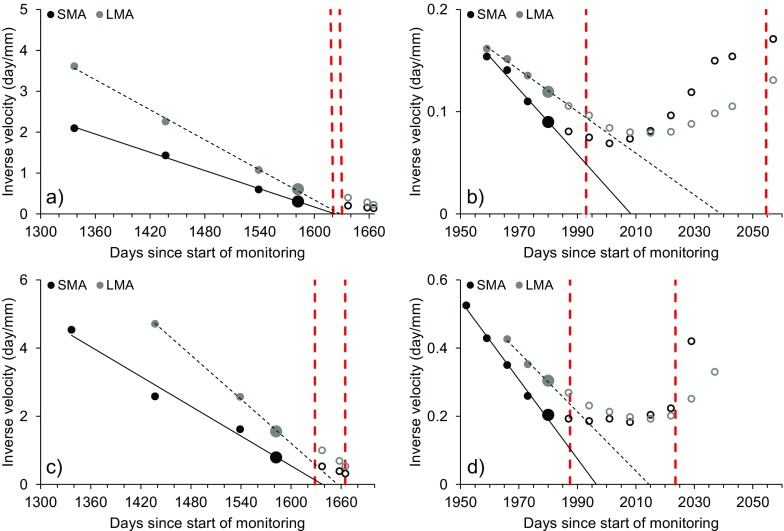
Failure window approach applied to inverse velocity data of pile 6. **a** First acceleration phase, horizontal direction. **b** Second acceleration phase, horizontal direction. **c** First acceleration phase, vertical direction. **d** Second acceleration phase, vertical direction. The red dashed lines define the limits of the failure window, the larger points mark days no. 1582 and 1980 (i.e., *T*_*c*_), and the hollow points represent measurements after *T*_*c*_

Figures 20 and 21 present the failure window analysis applied to monitoring data of pile 0 and pile 6, considering both acceleration phases and both directions of movement (i.e., horizontal and vertical). Extrapolation of the trend toward the time axis of data filtered by means of SMA and LMA was conducted by considering the last linear part of the plot up to the previously defined points of trend change. As mentioned, the latter were identified at the same time in the series for every pile (i.e., days no. 1582 and 1980 since start of monitoring). The number of data points before *T*_*c*_ that may be considered depends on the quality of the linear regression.

In every instance (i.e., for both acceleration phases and for both directions of movement), *T*_*c*_ occurred several days before the projected first limit of the failure window. Since at every point of trend change INV analyses should be restarted considering only data included in the new trend (Rose and Hungr 2007; Dick et al. 2014), the failure window plots in Figs. 20 and 21 represent the closest instants to a theoretical condition of failure of the 10-mile Slide for the available dataset.

In Tables 1, 2, 3, and 4, the results of the failure window analyses conducted for each pile at the peak of the acceleration phases are summarized. Concerning the movements in the horizontal direction, on average, the point of trend change (*T*_*c*_) anticipated the first *T*_*fw*_ limit by roughly 29 and 15 days in the case of the first and second acceleration phase, respectively. As for the movements in the vertical direction, these showed that on average *T*_*c*_ occurred roughly 31 and 9 days before the beginning of the failure window in the case of the first and second acceleration phase. The failure prediction analysis therefore provided similar results regardless of the considered direction of pile motion. The smaller distance between *T*_*c*_ and the first *T*_*fw*_ limit in the case of the vertical movements of the second acceleration phase may be attributed to the higher amount of data noise, which leads to an increase in failure prediction uncertainty and therefore to a larger width of the projected failure windows.

**Table 1:** Summary of failure window analyses for the horizontal movements of the central piles at the peak of the first acceleration phase (i.e., at point of trend change)

Pile	Start *T*_*fw*_ (no. of days)	End *T*_*fw*_ (no. of days)	*T*_*fw*_ duration (days)	Start *T*_*fw*_ − *T*_*c*_ (days)
0	1629	1630	1	47
1	1616	1624	8	34
2	1604	1640	36	22
3	1605	1640	35	23
4	1605	1618	13	23
5	1609	1635	26	27
6	1617	1627	10	35
7	1602	1632	30	20
8	1627	1637	10	45
9	1599	1640	41	17
Average	1611	1632	21	29

**Table 2:** Summary of failure window analyses for the horizontal movements of the central piles at the peak of the second acceleration phase (i.e., at point of trend change)

Pile	Start *T*_*fw*_ (no. of days)	End *T*_*fw*_ (no. of days)	*T*_*fw*_ duration (days)	Start *T*_*fw*_ − *T*_*c*_ (days)
0	1997	2059	62	17
1	1993	2056	63	13
2	1993	2055	62	13
3	1993	2055	62	13
4	1994	2053	59	14
5	1992	2055	63	12
6	1993	2055	62	13
7	1995	2055	60	15
8	1998	2047	49	18
9	2000	2046	46	20
Average	1995	2054	59	15

**Table 3:** Summary of failure window analyses for the vertical movements of the central piles at the peak of the first acceleration phase (i.e., at point of trend change)

Pile	Start *T*_*fw*_ (no. of days)	End *T*_*fw*_ (no. of days)	*T*_*fw*_ duration (days)	Start *T*_*fw*_ − *T*_*c*_ (days)
0	1600	1624	24	18
1	1604	1641	37	22
2	1609	1651	42	27
3	1615	1658	43	33
4	1609	1655	46	27
5	1610	1656	46	28
6	1629	1665	36	47
7	1636	1648	12	54
8	1614	1642	28	32
9	1605	1634	29	23
Average	1613	1647	34	31

**Table 4:** Summary of failure window analyses for the vertical movements of the central piles at the peak of the second acceleration phase (i.e., at point of trend change)

Pile	Start *T*_*fw*_ (no. of days)	End *T*_*fw*_ (no. of days)	*T*_*fw*_ duration (days)	Start *T*_*fw*_ − *T*_*c*_ (days)
0	1986	2037	51	6
1	1985	2036	51	5
2	1988	2028	40	8
3	1990	2025	35	10
4	1990	2018	28	10
5	1986	2025	39	6
6	1988	2024	36	8
7	1990	2025	35	10
8	1993	2027	34	13
9	1994	2028	34	14
Average	1989	2027	38	9

## Discussion and conclusions

The 10-mile Slide, part of the ancient Tunnel earthflow (Bovis 1985), has been active for several decades and has required regular maintenance of a highway and of a CN railway track that cross the active area. Since 2013, upslope retrogression of the landslide involving the area occupied by the CN railway track was observed. Detailed monitoring of the railway retaining pile wall, undertaken since February 2011, permitted to analyze the retrogression of the landslide and to assess characteristics and evolution of the displacement trends within and in proximity of the unstable area. Relatively to the monitoring period, total horizontal displacements between 2.5 and 4.5 m were measured for the central piles, with respective vertical displacements between 1.5 and 2.1 m. Since the 10-mile Slide showed phases of progressive acceleration (with values of horizontal velocity of the central piles between 10 and 18 mm/day at the peak of the second acceleration phase), predictions based on inverse velocity were also performed to determine the risk of a catastrophic failure. In particular, the recently proposed “failure window” approach (Carlà et al. 2016) was used in order to account for the implicit uncertainty of INV analyses and to evaluate, in retrospect, its suitability to the management of landslide accelerations that ultimately did not evolve to failure.

Pile displacements were validated by inclinometer data relative to the period 8 to 28 September 2015, and therefore were considered representative of the landslide movements. This was expected given that the 10-mile Slide is a translational landslide over a discrete, basal zone of shear that is not intercepted by the piles, with no differential movement within the moving mass. Moreover, the direction of resultant displacement of the piles has been observed to be consistent with the dip angle of the slip surface (Fig. 15a), indicating that the piles move downslope together with the landslide and that a significant component of rotation may be excluded. In January 2015, the piles of the wall within the area of retrogression (central piles, i.e., piles 0–9) started to accelerate. This first acceleration phase, reflecting the natural dynamics of the landslide, evolved to a steady state of constant deformation (3–9 mm/day and 2–4 mm/day in the horizontal and vertical directions, respectively), which lasted for roughly 1 year and was then followed by a second and more intense phase of progressive acceleration that was driven by the drilling activities related to the installation of 253 new shear piles. Subsequently, landslide movements decreased, and at the end of September 2016, as such stabilization works were being completed, significant displacements of the piles were no longer measured.

Monitoring data were analyzed in terms of the characteristics of the displacement vectors. As a result, two markedly different deformation behaviors were identified: the central piles (Type 1 behavior) displayed higher amounts of total displacement, associated with phases of progressive acceleration and a mostly constant direction of movement (i.e., consistent azimuth and dip angle of movement); in general, the lateral piles (Type 2 behavior) showed instead significantly lower total displacements, lack of phases of progressive acceleration, and an erratic-measured direction of movement (i.e., high variability of azimuth and dip angle of movement). The fact that few of the lateral piles on the eastern side of the wall (especially pile 15) showed a transition from Type 2 to Type 1 deformation behavior in the latest stage of the monitoring period suggests that the 10-mile Slide may be subject to additional retrogression beyond its current boundaries. Moreover, it was observed that, in the case of pile 15, stabilization of the azimuth angle of movement anticipated the progressive increase in velocity by approximately 3 months (Fig. 18). This further highlights the importance of performing deep analysis of displacement vectors in order to improve the understanding of how the landslide is evolving.

Several authors have proposed that the type of trend in inverse velocity plots is related to the nature of the underlying physical mechanism driving the instability (Petley and Allison 1997). In particular, a linear trend to failure has been deemed to occur when processes of stress-transfer during crack nucleation and growth are dominant (Main et al. 1993; Kilburn and Petley 2003), whereas asymptotic forms of inverse velocity plots (as evidently in the case of the retrogression of the 10-mile Slide) may be associated to plastic deformation over a reactivated slip/shear zone or surface (Petley et al. 2002). This gives further support to the conclusion that the 10-mile Slide represents a partial reactivation of the larger Tunnel earthflow along a pre-existing slip/shear surface.

In retrospect, application of the failure window approach resulted successful for both the “natural” slope acceleration (i.e., first acceleration phase) and the “anthropogenic” slope acceleration (i.e., second acceleration phase, which was related to the ongoing stabilization works). In fact, the method did not produce false alarms, and it indicated that the two acceleration phases of the 10-mile Slide did not develop up to a point close to a condition of theoretical failure. In every instance, the point of trend change for the displacement of the piles anticipated the onset of the failure window by several days (Figs. 20 and 21; Tables 1, 2, 3, and 4). The most critical predictions are provided by the vertical movements during the second acceleration phase; these, despite being affected by a higher level of noise and thus being less reliable than horizontal data, showed an average difference of 9 days between *T*_*c*_ and the start of the projected *T*_*fw*_. While it is true that *T*_*c*_ of the first acceleration phase was detected on hindsight, that is most likely due to the low frequency of measurements during that period (Figs. 19 and 20). On the other hand, the second acceleration phase was characterized by a higher frequency of measurements, and consequently, *T*_*c*_ was more evident (Fig. 21). Therefore, a high frequency of measurement (ideally daily or even near real time) is necessary at least during phases of landslide acceleration.

For reference Crosta and Agliardi (2002), after calibrating theoretical velocity curves leading to a failure of the Ruinon rockslide, defined threshold points of 30, 15, and 7 days before failure derived from INV analyses in order to activate respectively a state of pre-alert, alert, and emergency. Even if the failure window approach is more conservative than the classic inverse velocity method, it is observed that the first acceleration phase of the 10-mile Slide hardly exceeded the 30-day threshold, whereas the second acceleration phase in general produced results comprised between the 15- and the 7-day threshold proposed by Crosta and Agliardi (2002). This observation strengthens the conclusion that a failure of the 10-mile Slide should have rightly not been expected during the monitoring period and suggests that the method can be applied at this landslide and to landslides of similar behavior, granted that high frequency of displacement measurements is obtainable and that many false alarms are avoided. As the 10-mile Slide may be subject to further reactivation and retrogression, it is of critical importance that monitoring of the displacements of the piles continues also in the future.

The paper presents a case study that illustrates the use of measured displacements to evaluate the behavior of landslides and their potential for further retrogression and failure. In this regard, thorough insights may be gained by monitoring the deformation of structures built on the instability. The results show that a comprehensive analysis of the characteristics of slope surface deformation is a crucial source of information for determining the mechanism and evolution of landslides and for assessing the level of risk posed by phases of slope acceleration.
